# Cannabis Use and Its Impact on Mental Health in Youth in Australia and the United States: A Scoping Review

**DOI:** 10.3390/epidemiologia5010007

**Published:** 2024-02-29

**Authors:** Aayush Baral, Fahad Hanna, Ritesh Chimoriya, Kritika Rana

**Affiliations:** 1 Public Health Program, Department of Health and Education, Torrens University Australia, Melbourne, VIC 3000, Australia; baralaayush538@gmail.com (A.B.); fahad.hanna@torrens.edu.au (F.H.); 2 Philanthropy Nepal (Paropakari Nepal) Research Collaboration, Auburn, NSW 2144, Australia; r.chimoriya@westernsydney.edu.au; 3 School of Medicine, Western Sydney University, Campbelltown, NSW 2560, Australia; 4 Concord Institute of Academic Surgery, Concord Repatriation General Hospital, Concord, NSW 2139, Australia; 5 Translational Health Research Institute, Western Sydney University, Campbelltown, NSW 2560, Australia

**Keywords:** adolescent, cannabis use, substance abuse, mental health, marijuana, psychosis, depression, dependence, scoping review

## Abstract

Cannabis is a widely used substance among the youth population, with an estimated 2.8% currently smoking cannabis. Its popularity is growing due to the perception of its harmless nature and lack of dependence. However, this increase in use has been linked to mental health issues, especially since its partial decriminalisation in some part of the United States and Australia. The objective of this scoping review was to investigate the mental health impact of cannabis use among young people in Australia and the United States. A scoping review was conducted according to the Joanna Briggs Institute (JBI) protocol, and articles were searched from ProQuest Central and EBSCO Host (MEDLINE and CINAHL databases). A total of 24 articles were analysed, including systematic reviews, meta-analyses, and cohort, longitudinal, and cross-sectional studies. The findings indicate that cannabis use is associated with depression, psychosis, suicide, cannabis use disorder, dependence, decline in cognitive function, and the development of externalising behaviour, particularly attention deficit hyperactivity disorder. However, the relationship between cannabis use and anxiety is equivocal. Mental health issues were more prevalent with increased frequency, duration, intensity, and type of use. Female, minority, LGBTQI, African American, Aboriginal, and Torres Strait Islander youth and the age of onset of cannabis use were significant factors for the development of mental health problems. The increasing prevalence of cannabis use among high school and college students suggests the need for intervention by teachers, parents, and community health professionals to make them aware of its potential negative mental health outcomes. Moreover, policy-level interventions by the government are required to discourage young people from using cannabis.

## 1. Introduction

Cannabis is a substance that has been used for centuries and is estimated to be currently used by around 2.8% of the young population worldwide, making it increasingly popular among youth [1,2]. In the United States, cannabis is the second-most used substance after alcohol, with an estimated 7.1% of youth using it [3]. In addition, cannabis is the most used substance among Australian youth, with around 34.8% of Australians aged 14 years and above using it at least once in their lifetime [4]. Among young Australian males of the Aboriginal and Torres Strait Islander population, this percentage increases to about 70%, and among females, it is 20% [5]. These numbers appear to be increasing, especially after the legalisation of cannabis for recreational use since 2012 [6,7]. The most common reasons for cannabis use among these groups are a decreased perception of harm, dependence, or addiction [8].

While cannabis has medicinal value for various conditions, including epilepsy, multiple sclerosis, Parkinson’s disease, arthritis, and anxiety, anecdotal evidence should not overshadow assessments of its immediate or long-term harm, especially concerning age of onset and chronic exposure [9,10]. The youth population, especially the adolescent group, is more prone to using cannabis because they go through several physical as well as psychological changes, which increase their vulnerability to environmental influences, involvement in risk-taking behaviour, peer pressure, family relations, and conflicting and variable views of cannabis use by medical, political, social, and ethical standpoints on cannabis-related issues [5,11,12]. In addition, self-control behaviour by adolescents against cannabis use is also an important predictor of the development of cannabis use disorders or other mental health issues [13]. It has been found that young people are equally susceptible to cannabis use, with both males and females being equally susceptible, but males are more prone to its chronic use, whereas females are more susceptible to developing mental health problems [14]. Among numerous strains of cannabis, Cannabis sativa is the most popular and widely used, derived from leaves or female flower buds. The common mode of delivery of herbal cannabis is usually through inhalation of smoke [5] and, in recent days, cannabis use with vaping has increased substantially [15]. The psychoactive compound, i.e., 9 delta tetrahydrocannabinol (THC) present in cannabis, is responsible for most of its effects, which are mediated through the CB1 receptor present in the brain and are responsible for the development of various mental health conditions such as psychosis, depression, cannabis use disorders, dependence, anxiety, mood disorders, and other mental health problems [5]. Another active component found in cannabis is cannabichromene (CBC), which interacts with the endocannabinoid system in ways that suggest potential anti-inflammatory, pain-relieving, and neuroprotective effects, but its impact on mental health is not yet clearly understood [16].

The selection of Australia and the US for this study reflects the similarities in the intricate and dynamic nature of cannabis legalisation in these regions. With certain states having already legalised cannabis for both recreational and medicinal use, and others still in the process of considering it, these two countries present a unique context for examining the effects of cannabis on the mental health of young individuals [17,18,19]. However, there is limited research conducted specific to young people in Australia and United States that explores the impact on mental health, which have a similar landscape in terms of variation of legalisation in state and federal level. Despite the growing concerns surrounding the mental health implications of cannabis use among young people, there is a noticeable lack of reviews that comprehensively assess this issue. To address this knowledge gap, we conducted a scoping review aimed at examining and synthesising the existing evidence on the mental health impact of cannabis use among young populations in Australia and the United States.

## 2. Materials and Methods

This scoping review was conducted according to the protocol that was prepared based on to the Joanna Briggs Institute (JBI) protocol for scoping reviews [20]. This protocol included specific steps for identifying and selecting relevant studies, extracting data, and analysing and reporting the results to answer the research question.

### 2.1. Research Question

What is the nature and extent of the mental health impact of cannabis use among young people in Australia and the United States?

### 2.2. Eligibility

Using modified versions of the Population, Interventions, Comparators and Outcomes (PICO) framework, we formulated the research question and selected the eligibility criteria for the study. Peer-reviewed journal articles published in the English language and conducted on human subjects were screened as per eligibility criteria, which are summarised in Table 1.

### 2.3. Search Strategy, Information Source, and Study Selection

The search strategy employed in this scoping review involved the databases ProQuest Central, CINAHL (via EBSCOhost), and MEDLINE (via EBSCOhost). The initial search was conducted using words found in the title and abstract of retrieved papers, as well as index terms used to describe the articles. Following this, a second search was conducted using the identified keywords and index terms across all databases, incorporating limiters such as article publication dates between 1 January 2012 and 1 November 2022, age range between 12 and 45 years, peer-reviewed studies conducted in English language, studies with human subjects, and studies from the United States and Australia.

Keywords employed in the search strategy included the following:

- Mental health: Anxiety OR depression OR bipolar mood disorder OR psychosis OR schizophrenia OR attention deficit hyperactive disorder OR ADHD OR PTSD OR Anti-sociality disorder OR conduct Disorder OR Panic attack OR Mental disorder OR Mental*;
- Cannabis: Marijuana OR Tetra hydro cannabidiol OR THC OR pot OR bong OR joint OR cannabis;
- Young population: Adolescence OR adolescent OR young adult OR youth OR young.

All the identified studies were uploaded to Mendeley Version 2.8 (Elsevier, Amsterdam, The Netherlands), a reference management tool, which was used to remove duplicates, and to screen and select studies for the data extraction process, as per PRISMA Extension for Scoping Reviews [21]. Two reviewers (A.B. and K.R.) independently assessed the titles and abstracts of the articles and determined their eligibility based on the criteria provided in Table 1. The studies that met the criteria were retrieved in full text and screened. Any disagreement was resolved through consensus or discussion with a third reviewer (R.C.).

### 2.4. Data Extraction

Data extraction was carried out following the Preferred Reporting Items for Systematic Review and Meta-Analysis extension for Scoping Reviews (PRISMA-ScR) Checklist [21]. All the data were collected electronically and was extracted in a pre-developed form using Microsoft Word version 16.7 software. The form was calibrated and pilot-tested by two reviewers (A.B. and K.R.), and all the items collected were verified by a third reviewer (R.C.). Disagreements were resolved by consensus and consultation with all reviewers. The extracted data included the author’s name, year of publication, study design, information about participants, study objective, and study outcomes.

### 2.5. Thematic Analysis and Charting

After completing the data extraction process following the PRISMA-ScR checklist, we conducted thematic analysis and charting to analyse and summarise the findings [21]. Thematic analysis involves identifying, analysing, and reporting the themes found in the data, while charting involves summarising the key findings of each article and organising them to facilitate comparison and synthesis of results. Through this process, we were able to identify the key themes and patterns in the data and synthesise the findings from the different studies [22]. This helped provide a broader understanding of the mental health impact of cannabis use among young people in Australia and the United States.

## 3. Results

**Figure 1:**
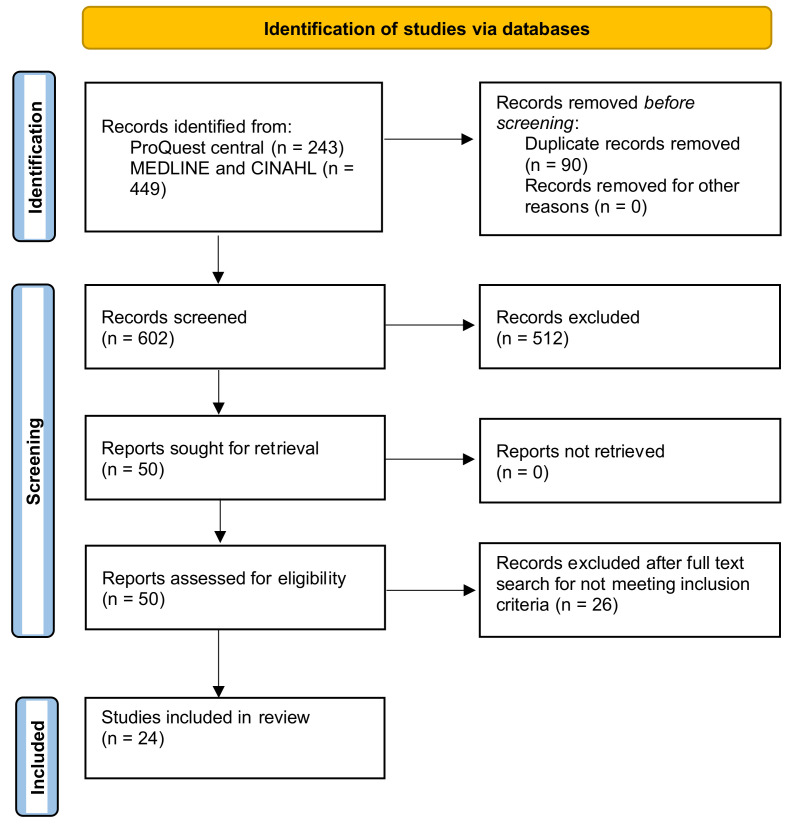
PRISMA flow diagram illustrating identification of studies.

The initial search retrieved a total of 692 articles from the electronic database search, as illustrated in Figure 1. Of these, 243 were from ProQuest Central, while 449 were obtained from EBSCOhost using 2 databases, which included CINAHL and MEDLINE, covering the period from 1 January 2012 to 1 November 2022. After removing 90 duplicates, 50 full-text studies were assessed, and 26 studies were subsequently excluded based on the eligibility criteria.

Out of the 24 articles included for data charting and thematic analysis, 3 were from Australia and 21 were from the United States, as shown in Table 2. Among them, three were systematic reviews and meta-analyses, seven were cohort studies, seven were longitudinal studies, and seven were cross-sectional studies.

### 3.1. Cannabis Use among Young Population and Depression and Depressive Symptoms

The relationship between cannabis use among young people and the development of depression and depressive symptoms was explored in 15 selected studies. A 2019 systematic review and meta-analysis conducted by Knopf et al. in the United States found 11 relevant articles, constituting individuals aged between 12 and 32 years, and concluded that teen cannabis users had 1.37 times higher risk of developing depression than non-users [25]. A similar trend was synthesised by another systematic review [41]. A cohort study conducted in Australia, consisting of participants from adolescence to young adulthood, found that weekly cannabis users were more prone to developing depression by 0.24 (95% CI: 0.18–0.30) compared to non-users and the strength of association was strongest among adolescents [27].

Moreover, cross-sectional research conducted in Baltimore, United States that included female participants of African American and white ethnicity aged between 18 and 30 years found that African American females were more likely to develop depression, with an odds ratio of 2.1 (95% CI: 1.08–3.93), and the relationship between cannabis use and depression did not differ by developmental stages [28]. A longitudinal study conducted in Australia among adolescents from age 15 to 19 years found that the rate of cannabis use increased from 7.5% at age 15 to 29.8% at age 19, which was four times more, and showed a 20% increase in depression between these ages [29]. A similar trend in the increase of cannabis use from adolescence to adulthood was found in the United States in one longitudinal research study conducted among 9816 individuals aged between 18 and 32 years [30]. However, there was an inverse relationship between the prevalence of depressive symptoms and the age of cannabis use, i.e., more among adolescents and less in emerging adulthood [29,30].

The frequency of cannabis use and the development of depression was directly related and twice as high among youth and did not significantly differ by age, as found in one cohort study conducted among 55,271 adolescents and young adults in the United States [31]. Depression was more common among regular non-blunt cannabis users as compared to blunt users in a cross-sectional study comprising participants from age 12 to 45 years [32]. However, one cohort study consisting of high school students and one longitudinal study consisting of young people in the United States found association with the development of depression in early adulthood, especially from 18–26 years [35,36]. This finding was consistent with another cross-sectional research conducted in 300 university students aged 18 to 25 years in Colorado, United States [38].

A longitudinal research study conducted in the United States from participants aged 12–32 years attempted to find the causal relationship between depression and cannabis use and found that it was bidirectional and more common among sexual minority groups such as LGBTQI groups [37]. This finding was supported by another cross-sectional study conducted in the United States consisting of 204,102 individuals aged 12–17 years [14].

### 3.2. Cannabis Use among Young Population and Anxiety

The relationship between cannabis use and anxiety in young people was explored in eight articles in this review. However, the results seem equivocal. For example, longitudinal research conducted among Australian secondary school students transitioning to young adulthood found that those who used cannabis were 20–30% more likely to have anxiety [29]. Another cohort study conducted in the United States with a sample of 55,271 individuals aged 12–32 years found that young people who used cannabis were twice as likely to develop anxiety, with the likelihood increasing with the intensity of use [31]. This finding is consistent with a longitudinal study conducted in the United States among individuals aged 18–45 years, with the highest prevalence observed in those aged 18–22 years [31,35]. Furthermore, a longitudinal study conducted in the United States with participants aged 12–32 years found that the relationship between cannabis use in adolescence and the development of anxiety in late adolescence/early adulthood is unidirectional and is more common among sexual minority groups such as LGBTQI individuals [37]. The relationship with cumulative cannabis use, regardless of status, was significant among young people aged 15–26 years in the United States [43].

However, a 2019 systematic review and meta-analysis that included 11 relevant articles found no statistically significant relationship between cannabis use among young people and the development of anxiety in the United States [41]. This finding was supported by a cross-sectional study conducted among university students aged 18–25 years in Colorado, United States [38]. Additionally, one longitudinal study consisting of 506 adolescent boys from Pittsburgh, United States, did not find a statistically significant outcome for depression among participants in their mid-30s across different subgroups of cannabis users, including early onset chronic users, late increasing users, adolescence-limited users, and low/non-users [33].

### 3.3. Cannabis Use among Young Population and Risk of Suicide, Suicidal Ideation, Plan, and Attempt

A study among high school students in the United States found that adolescents reporting a history of cannabis use were 2 to 5 times more likely to have had suicidal ideation, suicidal thought, and a suicidal plan in the last year [26]. This finding was also supported by a cohort study with a nationally representative sample in the United States [36]. A US cohort study on participants aged 18–34 found that past-year cannabis use disorder, daily cannabis use, and non-daily cannabis use were linked to higher rates of suicidal ideation, plan, and attempt [42]. The increase in prevalence was more significant among individuals with or without cannabis use disorder, with a higher prevalence observed among women (13.9% vs. 3.5%) than men (9.9% vs. 3.0%) [42]. Similarly, a systematic review and meta-analysis in 2019 found that teen cannabis users had a 1.5 times increased risk of developing suicidal ideation and were 3.46 times more likely to attempt suicide than non-users [41]. However, a longitudinal study consisting of adolescent boys from Pittsburgh, United States did not find a statistically significant outcome for suicide, suicidal plan, attempt, and ideation among participants in their mid-30s between different subgroups of cannabis users, i.e., from early onset chronic users, late increasing users, adolescence-limited users, and low/nonusers [33].

### 3.4. Cannabis Use among Young Population and Risk of Psychosis and Psychotic Symptoms

A longitudinal study conducted in the United States with 36,309 participants between the ages of 18 and 45 years found that the frequency of cannabis use and cannabis use disorder is directly proportional to the development of psychosis, with more prevalence between the ages of 18 and 23 and higher risk among females than males [35]. Additionally, a cohort study conducted in Boston, United States participants between the ages of 14 and 18 years found that cannabis use and the association of psychotic symptoms such as hallucination and paranoia are more common among youth with positive monthly or more frequent use of cannabis or cannabis use disorder as compared to use once or twice in the past year. However, cannabis use was also positively associated with psychotic symptoms, even with a single use [39].

### 3.5. Cannabis Use among Young Population and Risk of Cannabis Use Disorder and Dependence

A cohort study conducted in the United States with 55,271 participants aged between 12 and 45 years found that cannabis use disorder was directly proportional to the intensity of use. The disorder was more prevalent in heavy users (22.35%) compared to moderate (15.54%) and light users (10.10%). Additionally, recent users were more likely to report cannabis use disorder compared to non-users, with prevalence rates of 15.32% for the past 30 days, 11.62% for the past year, and 3.49% for lifetime use [31]. Similarly, a cohort study in the United States consisting of 281,650 individuals aged between 18 and 34 years found that individuals with past-year cannabis use disorder were associated with a higher prevalence of suicidal ideation, plan, and attempt [42]. Furthermore, another cohort study conducted in the United States found significant cannabis use disorder among participants, with 40% meeting the DSM IV criteria for cannabis dependence and 32% meeting the criteria for cannabis abuse. Additionally, adolescents with cannabis use disorder were more likely to develop alcohol and opioid abuse [8]. In addition to this, a cross-sectional research study conducted in the United States among participants aged between 12 and 45 years to determine the prevalence of cannabis use disorder and dependence among participants taking cannabis in different forms found that cannabis use disorder, abuse, and dependence increased most in blunt use, followed by dual cigar and blunt use, then non-blunt marijuana use [32]. A cross-sectional study conducted in the United States among 182,722 participants aged between 18 and 22 years found that there was an annual increase in marijuana use from 2002 to 2018 among college and non-college individuals by 0.46% vs. 0.37%, respectively. However, there was no statistically significant result for the development of cannabis use disorder [24].

### 3.6. Cannabis Use and Development of Other Mental Disorder

A meta-analysis with more than 650 study subjects and 5600 controls found that cannabis use was associated with the development of cognitive decline, as measured by a decrease in IQ by 2 points compared to non-users in the United States [40]. Another cohort study conducted in Boston, United States with participants between the ages of 12–24 years found that cannabis use was associated with an increased risk of developing externalising behaviours, especially attention deficit hyperactivity disorder, compared to non-users [34].

## 4. Discussion

This scoping review systematically synthesised evidence on the knowledge gaps in understanding the relationship between cannabis use among young individuals and its impact on mental health. Analysing 24 articles primarily from the United States and Australia, it revealed a consistent association between cannabis use and various adverse mental health outcomes, including depression, anxiety, suicide, psychosis, cannabis use disorder, dependence, lower cognitive function, and externalising behaviour. A consistent association between cannabis use and depression was synthesised from the included studies, and, additionally, we observed a higher prevalence of cannabis use among sexual minority groups, including LGBTQI youth, African American youth, and Aboriginal and Torres Strait Islander youth populations [5,13,28,37]. Such a trend can be explained through the mental health stigma and drug-related stigma in marginalised groups [44,45]. Nevertheless, cannabis use exhibited consistency between male and female youth, with males displaying a higher likelihood of chronic use, while females were more prone to developing adverse mental health outcomes [23,28,30,35]. Furthermore, non-blunt cannabis use was more consistently associated with the development of depression than blunt use, and adolescents and young adults aged between 18 and 26 years were found to be more susceptible to developing depression from cannabis use [32]. Previous global-scale studies have noted similar tendency as well [46]. The findings regarding the development of anxiety among young adults were ambivalent, as anxiety was directly related to intensity, past cumulative use, and age group. Additionally, it was more commonly observed among sexual minority groups [27,31,35,37,41]. Cannabis use was associated with the development of psychosis and psychotic symptoms, such as hallucinations and paranoia [35,39]. Cannabis use is associated with heightened risks of cannabis use disorder, suicide, substance problems, particularly among females and males, respectively, with conflicting findings possibly due to study limitations, while also correlating with decreased cognitive capacity and externalising behaviour [34,40].

The established relationship between cannabis use and a spectrum of mental health outcomes presents a discourse with significant implications for diverse stakeholders and particular relevance for medical practitioners, policymakers, and mental health professionals, including developmental psychologists [47,48,49]. Integrating this research into practice could offer valuable insights for medical practitioners, prompting us to consider cannabis use as a pertinent factor when addressing mental health issues in young people [50,51]. Furthermore, the age groups, gender differences, and vulnerable populations underscore the need for tailored interventions and preventive strategies demanded by the changing demographics of the population [52,53,54,55,56]. However, the critical discussion should acknowledge potential limitations within the research, such as variations in study methodologies and the influence of biases and confounders. This underscores the necessity for continued investigation and the application of an evidence-based approach in both clinical and policy settings to address the complex interplay between cannabis use and mental health outcomes [57].

The findings underscore the need for future research to focus on longitudinal studies examining the temporal relationship between cannabis use and mental health outcomes. This includes factors such as age of onset, frequency, and duration of use, as well as the impact of different strains, modes of delivery, and dosages on mental health [53,54,55,56]. Building on this research, policies should be evidence-based, balancing potential benefits with risks, with stricter regulations on availability and targeted interventions for vulnerable groups [56,57,58]. Prevention efforts should include comprehensive school-based programs to educate students about the risks of cannabis use and promote healthy coping strategies [59]. Healthcare provider training is also crucial to ensure professionals can screen for cannabis use, provide early interventions, and offer support to at-risk individuals [60]. Educational programs should aim to dispel misconceptions and stigma, while providing accurate information and promoting healthy coping strategies [59,60].

Most of the studies were observational studies, which can be affected by unmeasurable confounders. Therefore, future research needs to be more robust, such as systematic reviews and meta-analyses, translational qualitative studies, and controlled trials, to understand the causal relationship between cannabis use and the development of mental illness among young populations by adjusting potential non-measurable confounders [61,62]. Furthermore, the increasing prevalence of cannabis use among high school and college students, due to its perceived harmless nature and lack of dependence, suggests the need for intervention by integrating teachers, parents, courses, and community health professionals to make them aware of its potential negative mental health outcomes [63,64]. Additionally, policy-level interventions to discourage cannabis use among young populations are necessary [65]. Overall, this review emphasises the importance of understanding the potential mental health consequences of cannabis use among young people and suggests the need for more robust research and intervention strategies to prevent these outcomes.

This scoping review was conducted through a search of three databases from 2012 to 2022. Nevertheless, it is important to acknowledge certain limitations inherent in this approach. The reliance on three specific databases might result in overlooking relevant articles available on alternative platforms and the gray literature, potentially leading to a fractional representation of the existing literature. Furthermore, given that a majority of the included articles were observational studies, the research is susceptible to recall bias, selection bias, and unmeasurable confounders, introducing potential threats to the study’s internal validity [58,66]. Additionally, confining the search to the United States and Australia may cause limitations about the generalisability of the findings to other global regions, which may exhibit distinct geographical, political, cultural, and societal variations impacting the relationship between cannabis use and mental health outcomes. Therefore, it is crucial to interpret the results within the context of these limitations and consider the need for further research in diverse settings to enhance the robustness and generalisability of the findings of this scoping review.

## 5. Conclusions

This scoping review was conducted to identify the mental health impact of cannabis use among the young populations of Australia and the United States. It was found that cannabis use has been associated with the development of depression, psychosis, cannabis use disorder (CUD), dependence, externalising behaviour, attention deficit hyperactivity disorder (ADHD), and a decline in cognitive function. However, there were equivocal results regarding the development of anxiety. Additionally, the development of these disorders was associated with the age of cannabis use, intensity, and type of use, and was more common among females and in vulnerable populations. Most of the studies reviewed were observational; hence, a causal relationship between cannabis use and mental health outcomes requires more robust forms of research.

## Figures and Tables

**Table 1:** Inclusion criteria adopted in the scoping review.

PICO Elements	Inclusion Criteria
Population	Young population aged 12–45 years (both male and female)
Intervention	Cannabis use
Comparison	N/A
Outcome	Mental health outcomes diagnosed using standard diagnostic tool (e.g., DSM III or IV)
Context	Australia and/or United States
Study Design	Primary research studies, systematic reviews and meta-analyses, prospective or retrospective cohort studies, case–control, randomised control trials, cross-sectional studies, longitudinal studies, and guidelines published between 1 January 2012 and 1 November 2022

**Table 2:** Summary of the included studies.

No.	Author and Year of Publication	Country	Sample Size and Population	Study Design	Aim/Objective	Summary of Findings
1	Moller et al., 2013 [23]	Australia	Sample size = 4160; aged 20–24 and 40–44.	Cohort	To investigate the factors that predict self-harm among Australian adults, with a focus on substance use and psychological distress.	Past-year self-harm was reported by 8.2% of participants (95% CI 7.4–9.0%), with 9.3% of males (8.0–10.6%) and 7.3% of females (6.2–8.4%) who reported current cannabis use.
2	McCabe et al., 2021 [24]	United States	Sample size = 182,722; aged 18–22 years	Cross-sectional	To evaluate national trends over time in past-year alcohol and marijuana abstinence, co-use, alcohol use disorder, and marijuana use disorder among young adults in the US, considering their college status (2002–2018).	There was a yearly rise in marijuana use among young adults from 2002 to 2018, both for those in college (0.46%; 95% CI, 0.37–0.55%) and those not in college (0.49%; 95% CI, 0.40–0.59%). However, there was no corresponding increase in marijuana use disorder among all young adults.
3	Knopf, 2019 [25]	United States	Sample size = 23,317 in 11 articles; aged 12–32 years	Systematic review and meta-analysis	To assess cannabis use among adolescents under 18 years old (at least one assessment point) and determine its association with the development of depression in young adulthood (ages 18 to 32).	Teen cannabis users had more than three times the risk of attempting suicide in young adulthood compared to nonusers (OR: 3.46). Additionally, they faced an increased risk of developing depression (OR: 1.37) and experiencing suicidal ideation (OR: 1.50).
4	Zaman et al., 2015 [8]	United States	Sample size = 483; aged 12–18 years	Cohort	To assess the rates of cannabis abuse and dependence among adolescents referred for substance use evaluations, as well as the incidence of co-occurring psychiatric illnesses and substance use disorders among those individuals.	Found that 47% of the participants met the criteria for the Diagnostic and Statistical Manual of Mental Disorders IV, and an additional 32% met the criteria for cannabis abuse. Among adolescents with cannabis use disorders, there was a high co-occurrence of alcohol and opioid abuse or dependence. These individuals also experienced significant psychiatric comorbidities.
5	Wong et al., 2013 [26]	United States	Sample size = 73,183; high school students aged between 12 and 15 years	Cross-sectional	To investigate the link between different patterns of substance use and suicidality among a nationally representative sample of high school students in the United States.	Adolescents with a history of marijuana use showed associations with suicidal ideation, suicide planning, suicide attempts, and severe suicide attempts in the past year, even after adjusting for other confounding factors (OR = 1.9–5.2).
6	Horwood et al., 2012 [27]	Australia	Sample size = 6900; aged 12–45 years	Cohort	To assess the association between the frequency of cannabis use and the severity of depressive symptoms using data from four Australasian cohort studies.	The frequency of cannabis use was significantly associated with increasing depressive symptoms (*p* < 0.001). After adjusting for confounders, the depression scores for weekly users were 0.24 (95% CI 0.18–0.30) standard deviations higher than those for non-users. This association was consistent across the cohorts studied. Adolescents showed a particularly increased association.
7	Floyd Campbell, 2018 [28]	United States	Sample size = 120; African American and 111 white females; aged 18–30 years	Cross-sectional	To assess how the connection between depression and marijuana use varies between females in emerging adulthood and young adult females.	Found that 35% (75) of the participants tested positive for cannabis, and 21% (48) were experiencing depression, with an odds ratio (OR = 2.1; 95% CI 1.08–3.93). African Americans had higher odds of testing positive for marijuana compared to whites (OR = 4.6; 95% CI 2.52–8.68). There was no difference in the relationship between depression and marijuana use based on developmental stage.
8	Scholes-Balog et al., 2013 [29]	Australia	Sample size = 927; young people at ages of 15, 16, 17 and 19 years	Cohort	To examine the changing rates of cannabis use, misuse, and cannabis-related social harms among Australian adolescents as they transition into young adulthood.	The rates of cannabis use increased with age, with past-year use rising from 7.5% at age 15 to 29.8% at age 19. Cannabis use was more common among males than females at ages 17 and 19. Among cannabis users, the rates of cannabis-related harms were moderate, with anxiety and depression being the most prevalent, affecting 20–30% of users at each age.
9	Wilkinson et al., 2016 [30]	United States	Sample size = 9816; aged 18–32 years	Cohort	To investigate the longitudinal associations between substance use frequency and depressive symptoms from adolescence into young adulthood, with a focus on potential moderation by sex.	The frequency of cannabis use increases from adolescence to young adulthood, aligning with the rise in adolescent depressive symptoms, and this increase is positively associated with depressive symptoms from adolescence to young adulthood, with a stronger association for females. The proportion of respondents reporting cannabis use increases from 14% at ages 14–16 years to 25% at ages 20–22 years, then decreases to 17% for ages 32–34 years. Depressive symptoms follow the opposite pattern, starting higher in adolescence, decreasing in emerging adulthood, and then increasing slightly in young adulthood. For instance, the mean CES-D score for ages 14–16 years is 5.72, decreasing to 4.28 at ages 23–25 years, but then increasing again to 5.08 between ages 29 and 31 years.
10	Richter et al., 2017 [31]	United States	Sample size = 55,271; adolescent and young adult (exact age not specified)	Cohort	To provide recent national estimates of cannabis use disorder based on usage patterns, age, and other sociodemographic, substance use, and mental health variables.	The prevalence of cannabis use disorder (CUD) was higher among heavy (22.35%) or moderate (15.54%) users compared to light users (10.10%), and about twice as high among youth compared to adults. Anxiety was associated with a higher prevalence of CUD among heavy marijuana users only, while depression showed no significant association. Current marijuana use at any level was linked to increased odds of CUD compared to no current use, with odds increasing with intensity of use (OR = 8.20 for light, OR = 13.61 for moderate, and OR = 23.59 for heavy users, all *p* < 0.002). Age of initiation, anxiety, and depression did not show significant associations with CUD. In 2014, 3.49% of lifetime, 11.62% of past-year, and 15.32% of past-30-day marijuana users met DSM-IV criteria for CUD, with rates among youth generally at least double those of adults.
11	Cohn et al., 2016 [32]	United States	Sample size = 54,309; aged 12–45 years	Cross-sectional	To identify subtype differences, this study examined the prevalence and correlates of four exclusive groups of users (cigar-only, blunt-only, non-blunt marijuana, or dual cigar–blunt) in terms of demographic, mental health, and substance use characteristics.	Those who reported using blunts only or both cigars and blunts endorsed a greater number of symptoms of cannabis use disorder (CUD) compared to those who reported using cigars only or non-blunt marijuana. Lower perceptions of marijuana risk were associated with increased odds of cannabis use, with or without blunts. Major depressive episode (MDE) was uniquely linked to non-blunt cannabis use. Participants who reported blunt-only (AOR = 9.80) and dual cigar–blunt use (AOR = 9.76) endorsed the highest number of CUD symptoms, followed by those who reported non-blunt marijuana use (AOR = 7.61) and cigar-only use (AOR = 2.55).
12	Bechtold et al., 2015 [33]	United States	Sample size = 506; 41.7% White, 54.5% Black, 3.8% other	Longitudinal	To assess whether distinct developmental trajectories of marijuana use, tracked annually from early adolescence to the mid-20s, were linked to negative physical (e.g., asthma, high blood pressure) and mental (e.g., psychosis, anxiety disorders) health outcomes in the mid-30s.	Latent class growth curve analysis revealed four distinct subgroups of marijuana users: early onset chronic users, late increasing users, adolescence-limited users, and low/nonusers. However, these groups did not significantly differ in terms of their physical and mental health outcomes, including anxiety and suicide problems, assessed in the mid-30s.
13	Welsh et al., 2017 [34]	United States	Sample size = 483; aged 12–24 years	Cohort	To examine the relationship between mental health diagnoses and substance use disorders in adolescents and explore differences between different mental health diagnoses and types of substances used.	Cannabis use was associated with the development of externalising behaviour disorders (OR = 2.10, *p* = 0.024). Youths with problematic use, abuse, or dependence on marijuana were 2.20 times as likely to have an externalising behavioural disorder and twice as likely to have attention deficit hyperactivity disorder (ADHD) (odds ratio = 2.10, *p* = 0.024).
14	Leadbeater et al., 2019 [35]	United States	Sample size = 36,309; aged 18 and above	Longitudinal	To assess the strength of the associations between cannabis use frequency, cannabis use disorder (CUD), and mental health symptoms.	More frequent cannabis use was associated with increased psychotic symptoms from age 18 (b = 0.21, 95% CI = 0.10, 0.33) to age 65 (b = 0.36, 95% CI = 0.16, 0.56), higher depressive symptoms from age 18 (b = 0.22, 95% CI = 0.05, 0.40) to age 64 (b = 0.25, 95% CI = 0.01, 0.48), and increased anxiety symptoms from age 20 (b = 0.07, 95% CI = 0.01, 0.13) to age 63 (b = 0.13, 95% CI = 0.01, 0.25). Cannabis use disorder (CUD) was associated with more psychotic symptoms from age 18 (b = 1.21, 95% CI = 0.66, 1.76) to age 64 (b = 1.09, 95% CI = 0.05, 2.12), more depressive symptoms from age 18 (b = 0.96, 95% CI = 0.19, 1.73) to age 61 (b = 1.11, 95% CI = 0.01, 2.21), and more anxiety symptoms from age 18 (b = 0.45, 95% CI = 0.02, 0.88) to age 62 (b = 0.75, 95% CI = 0.08, 1.43). Time-varying interactions of CUD with early age of onset were not statistically significant. Females exhibited stronger associations between CUD and mental health symptoms than males for psychotic symptoms between ages 18 and 23, depressive symptoms between ages 18 and 26, and anxiety symptoms between ages 18 and 22.
15	Chadi et al., 2019 [36]	United States	Sample size = 26,821; aged 12–18 years	Cohort	To investigate the association between e-cigarette and marijuana use and depressive symptoms and suicidality in a large, nationally representative sample of high school students.	Cannabis use was reported by 9.7% of participants and was associated with depression (AOR: 1.25, 95% CI 1.04–1.50) and suicidality (AOR: 1.49, 95% CI 1.27–1.75).
16	London-Nadeau et al., 2021 [37]	United States	Sample size = 1430; conducted in waves when age of the participants was 13, 15, and 17 years	Longitudinal	To investigate the relationships between cannabis use and symptoms of depression and anxiety at ages 13, 15, and 17 years.	Cannabis use at 13 and 15 years predicted anxiety symptoms at 15 and 17 years, while depression symptoms at 15 years predicted cannabis use at 17 years. Differences were found between heterosexual and LGBTQI participants. LGBTQI individuals showed a stronger association between depression symptoms at 15 years and cannabis use at 17 years, and a negative association between anxiety symptoms at 15 years and cannabis use at 17 years. This suggests bidirectional relationships between cannabis use and depression/anxiety symptoms in adolescence.
17	Weinberger et al., 2020 [14]	United States	Sample size = 204,102; aged 12–17 years	Cross-sectional	To investigate the association between depression and increased cannabis use among youth in the United States, both overall and by demographic factors. The study also aimed to examine trends in cannabis use by depression status among youth from 2004 to 2016.	Between 2004 and 2016, cannabis use rose among both youth with and without depression. However, the increase was significantly faster among youth with depression (8.45% to 11.65%) compared to those without depression (4.28% to 4.71%). Youth with depression were more than twice as likely to report cannabis use (12.86% versus 6.40%) compared to those without depression.
18	Phillips et al., 2018 [38]	United States	Sample size = 300; university students from 18–25 years	Cross-sectional	To examine whether marijuana use and related issues were associated with various demographic, personality, and psychological factors among college students at a mid-sized university in Colorado.	Out of 300 participants, 219 (73%) reported lifetime marijuana use, 195 (65%) reported use in the last year, and 126 (42%) reported use within the last 30 days. Approximately 29% of all participants (*n* = 88; 21% of females and 43% of males) tested positive for marijuana on the urine screen, confirming recent use. Among the three psychological factors (social anxiety, general anxiety, and depression), only depression was associated with cannabis use.
19	Levy and Weitzman, 2019 [39]	United States	Sample size = 527; aged 14–18 years	Cohort	To evaluate whether adolescents presenting for routine medical care had experienced acute psychotic symptoms during or immediately after marijuana use.	Overall, 27.4% of respondents reported hallucinations, 33.6% reported paranoia or anxiety, and 42.9% reported at least one symptom. None of hallucinations, paranoia, or anxiety correlated with age, sex, race/ethnicity, general health status, or socioeconomic status. However, respondents meeting criteria for cannabis use disorder were more likely to report these symptoms. Among respondents, 47.9% reported using marijuana “monthly or more” in the past year, and this group was more likely to report hallucinations and paranoia compared to those who used “once or twice” (60.0% vs. 40%).
20	Power et al., 2021 [40]	United States	Over 650 subjects and 5600 controls	Systematic review and meta-analysis	To explore whether cannabis has an impact on full-scale IQ in general population samples, contributing to a better understanding of this potential pathway.	Cannabis use in youth was associated with modest IQ differences, equating to approximately a two-point decrease in young cannabis users.
21	Gobbi et al., 2019 [41]	United States	A total of 11 studies comprising 23,317 individuals were included in the quantitative synthesis	Systematic review and meta-analysis	To quantify the association between cannabis use during adolescence and the risk of developing subsequent major depression, anxiety, and suicidal behaviour.	The OR for developing depression in young adulthood for cannabis users compared with nonusers was 1.37 (95% CI, 1.16–1.62; I2 = 0%). The pooled OR for anxiety was not statistically significant, at 1.18 (95% CI, 0.84–1.67; I2 = 42%). However, the pooled OR for suicidal ideation was 1.50 (95% CI, 1.11–2.03; I2 = 0%), and for suicidal attempt, it was 3.46 (95% CI, 1.53–7.84, I2 = 61.3%).
22	Han et al., 2021 [42]	United States	Sample size = 281,650; aged 18–34 years	Cohort	To assess whether cannabis use and cannabis use disorder (CUD) are linked to a higher prevalence of suicidality among young adults, with or without depression, and to assess if these associations differ by sex.	The adjusted prevalence of suicidal ideation, plan, and attempt increased 1.4 to 1.6 times from the 2008–2009 to 2018–2019 periods (adjusted risk ratio (ARR) for suicidal ideation, 1.4 (95% CI, 1.3–1.5); ARR for suicide plan, 1.6 (95% CI, 1.5–1.9); ARR for suicide attempt, 1.4 (95% CI, 1.2–1.7)). Past-year cannabis use disorder (CUD), daily cannabis use, and nondaily cannabis use were associated with a higher prevalence of past-year suicidal ideation, plan, and attempt in both sexes. Among individuals without major depressive episode, the prevalence of suicidal ideation for those with CUD was 13.9% vs. 3.5% among women and 9.9% vs. 3.0% among men; *p* < 0.001. The suicide plan among those with CUD and major depressive episode was 52% higher for women (23.7%) than men (15.6%); *p* < 0.001.
23	Pahl et al., 2014 [13]	United States	Sample size = 838; conducted in waves when participants were 14, 19, 24, and 29 years	Cohort	To examine adolescent self-control as a predictor of membership in joint developmental trajectories of depressive mood and marijuana use from adolescence to young adulthood.	The low marijuana use and low depressive mood (LL) group, reporting no marijuana use, consistently had the lowest depressive mood over time. The low marijuana use and intermediate depressive mood (LI) group, with minimal use in early adolescence and no use by age 29, showed higher depressive mood in early adolescence that decreased linearly over time. The high marijuana use and low depressive mood (HL) group, starting marijuana use early and increasing over time, reported depressive mood similar to the LL group. The high marijuana use and high depressive mood (HH) group, mirroring the HL group’s use pattern, reported the highest depressive mood in early adolescence, continuing into young adulthood. Thus, the co-occurrence of high marijuana use and depressive mood from adolescence to young adulthood is linked to low self-control in adolescence. Conversely, high self-control is associated with low marijuana use and depression over time.
24	Meier et al., 2020 [43]	United States	Sample size = 506; aged 15–26 years	Longitudinal	To determine whether increases in recent and cumulative cannabis use are associated with increases in internalising problems from adolescence to young adulthood.	After adjusting for time-varying covariates, the study found that increases in cumulative years of weekly cannabis use, but not recent use, were linked to higher levels of depression symptoms and anxiety/depression problems. Specifically, each additional year of prior weekly cannabis use was associated with a slight increase in depression symptoms (b = 0.012, *p* = 0.005) and anxiety/depression problems (b = 0.009, *p* = 0.001).

Abbreviations: AOR: Adjusted Odds Ratio; ARR: Adjusted Risk Ratio; CES-D: Center for Epidemiologic Studies Depression Scale; CI: Confidence Interval; CUD: Cannabis Use Disorder; DSM-IV: Diagnostic and Statistical Manual of Mental Disorders, Fourth Edition; I2: Measure of heterogeneity in meta-analysis; MDE: Major Depressive Episode; OR: Odds Ratio.
